# Biogenic silver nanoparticles from chamomile incorporated in hydrogels for high transparent non-infectiveness contact lenses

**DOI:** 10.1007/s00775-025-02121-0

**Published:** 2025-08-04

**Authors:** Panagiotis K. Raptis, Christina N. Banti, Christina Papachristodoulou, Sotiris K. Hadjikakou

**Affiliations:** 1 Biological Inorganic Chemistry Laboratory, Department of Chemistry, University of Ioannina, 45110 Ioannina, Greece; 2 Department of Physics, University of Ioannina, Ioannina, Greece; 3 University Research Center of Ioannina (URCI), Institute of Materials Science and Computing, Ioannina, Greece

**Keywords:** Biological inorganic chemistry, Silver nanoparticles, Chamomile extract, Antimicrobial materials, Hydrogels, Contact lens

## Abstract

Chamomile extract (CHA) was employed in the synthesis of silver nanoparticles (**AgNPs(CHA)**). These nanoparticles were incorporated into a hydroxyethyl methacrylate (pHEMA) matrix during polymerization at concentrations of 1 mg/mL (**pHEMA@AgNPs(CHA)_1**) and 2 mg/mL (**pHEMA@AgNPs(CHA)_2**). The resulting materials— **AgNPs(CHA)**, **pHEMA@AgNPs(CHA)_1**, and **pHEMA@AgNPs(CHA)_2** —were characterized using a plethora of analytical techniques, including Refractive Index (RI), X-ray fluorescence (XRF) spectroscopy, X-ray powder diffraction (XRPD), thermogravimetric-differential thermal analysis (TG–DTA), differential scanning calorimetry (DSC), ultraviolet–visible (UV–Vis) spectroscopy, and attenuated total reflection Fourier-transform infrared (ATR-FTIR) spectroscopy in the solid state, as well as UV–Vis spectroscopy in solution. XRPD analysis revealed a crystallite size of 8.02 nm, while the diameter of the nanoparticles, estimated using the λmax of the Surface Plasmon Resonance (SPR) spectrum, was 37 nm. The antimicrobial properties of the materials were evaluated against Gram-negative bacteria, *Pseudomonas aeruginosa* and *Escherichia coli*, as well as Gram-positive bacteria, *Staphylococcus epidermidis* and *Staphylococcus aureus*. These pathogens are commonly implicated in microbial keratitis. Antimicrobial efficacy was assessed using Minimum Inhibitory Concentration (MIC), Minimum Bactericidal Concentration (MBC), and Inhibitory Zones (IZs). The IZs for *P. aeruginosa*, *E. coli*, *S. epidermidis*, and *S. aureus* following incubation with paper discs soaked in 1 mg/mL of **AgNPs(CHA)** were 16.0 ± 0.6 mm, 11.1 ± 0.9 mm, 15.8 ± 1.2 mm, and 15.1 ± 0.2 mm, respectively. Bacterial viability after incubation with hydrogel discs of **pHEMA@AgNPs(CHA)_1** showed reductions to 0.9 ± 0.2%, 100.0 ± 0.0%, 1.9 ± 1.0%, and 4.4 ± 1.5% for *P. aeruginosa*, *E. coli*, *S. epidermidis*, and *S. aureus*, respectively. Additionally, no toxic effects were observed on human corneal epithelial cells (HCECs). These findings suggest that **pHEMA@AgNPs(CHA)_1** has potential as a promising candidate for developing non-infectious contact lenses.

**Supplementary Information:**

The online version contains supplementary material available at 10.1007/s00775-025-02121-0.

## Introduction

Biogenic nanomaterials, produced by living organisms, represent a highly promising class of materials with vast applications in fields such as medicine, biotechnology, and environmental engineering [1–3]. This is due to their unique and often superior properties compared to synthetic nanomaterials [1–3].

Silver nanoparticles (AgNPs) are utilized in various biomedical fields, including eye care, drug delivery, pharmaceuticals, orthopedics, surgery, and dentistry, among others [4]. Silver-containing biomaterials are already commercially available and are used in applications such as wound dressings, contraceptive devices, endotracheal tubes, bone prostheses, burn treatments, catheters, vascular grafts, surgical instruments, and dental devices [5]. Notably, silver-containing hydrogels enable AgNPs to interact with cellular membranes, demonstrating sensitivity toward bacterial membranes while exhibiting minimal effects on eukaryotic cell membranes [5]. The biological activity of AgNPs is closely linked to their particle size, with smaller particles exhibiting a stronger antimicrobial effect. Thus, engineered nanoparticles (NPs) with unique properties—such as their small size (< 100 nm), high surface-to-volume ratio, and enhanced reactivity—are highly promising candidates for use as anticancer and antimicrobial agents [6–8]. The mechanism of action of AgNPs resembles that of silver compounds [5]. AgNPs naturally interact with the surfaces of bacterial cells, particularly Gram-negative bacteria, where their adhesion and accumulation are commonly observed [9]. Research indicates that AgNPs can disrupt bacterial cell membranes, inducing structural changes that increase membrane permeability and ultimately lead to bacterial death [9]. Silver nanoparticles (AgNPs) have been synthesized using various methods, including physical, chemical, photochemical, microemulsion, biological, and microwave techniques [10]. A novel, environmentally friendly, high-yield, and cost-effective approach, known as biogenic synthesis, utilizes plant parts and microorganisms as capping agents [10]. Plant extracts derived from bark, stems, roots, leaves, flowers, oils, fruit peels, seeds, and seaweed, as well as microorganisms such as fungi, bacteria, and yeast, are employed in this method [10]. However, plants are a primary focus of research in this field due to their unique medicinal properties and their ability to produce AgNPs in a distinctive manner [10–12].

**Scheme 1:**
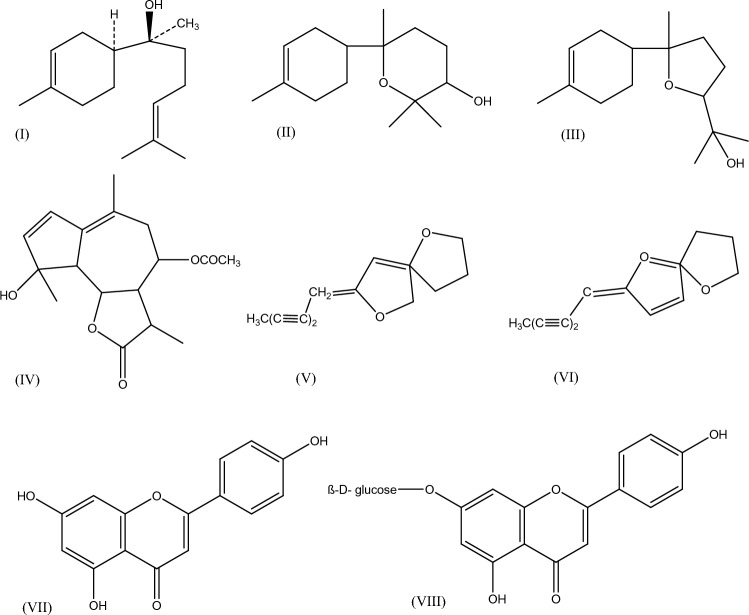
Chemical Structures of Chamomile extract main ingredients: (I). α-Bisabolol, (II). α-Bisabolol Oxide A, (III). α-Bisabolol Oxide B, (IV). Matricine, (V). cis-En-in-dicycloether, (VI). trans-En-in-dicycloether (spiroethers), (VII). Apigenin, (VIII). Apigenin-7-glucoside [13–15]

Chamomile flowerheads and extracts are used in the pharmaceutical and especially in the cosmetic industry. Its antimicrobial and antioxidant properties are already known [13, 14]. At present, many mouthwashes and sprays made with chamomile are used for oral bacteriostasis in clinical products [15]. The therapeutic and antioxidant properties, along with a significant portion of the pharmacological effects of chamomile, are attributed to its biologically active chemical components (Scheme 1) [13, 14]. The primary components of its extract are sesquiterpene compounds such as bisabolol, bisabolol oxides A and B, azulene, and phenolic compounds (Scheme 1). Among its various flavonoids, apigenin stands out as the most promising compound, along with other constituents like coumarins (Scheme 1) [14, 15].

Chamomile was selected as the stabilizing agent for the green synthesis of AgNPs due to its high content in flavonoids (e.g., apigenin, luteolin, quercetin), terpenoids (e.g., α-bisabolol), and phenolic acids, which are known to facilitate the reduction of silver ions and stabilize the resulting nanoparticles. Moreover, chamomile is a widely recognized medicinal plant with established anti-inflammatory, antimicrobial, and antioxidant properties, making it an attractive candidate for biomedical and ophthalmic applications. These intrinsic properties are expected to synergize with the antimicrobial action of AgNPs, potentially enhancing the biological activity of the hybrid material. Importantly, chamomile extract is biocompatible and well tolerated, which aligns with the safety requirements for ophthalmic use.

Microbial keratitis (MK), on the other hand, is a rare corneal infection. However, the likelihood of developing MK increases with contact lens use [16, 17]. Although microbial keratitis is relatively rare, the large number of soft contact lens users in the United States—approximately 45 million—leads to thousands of reported cases each year. [16, 17]. One approach to developing next-generation soft contact lenses involves incorporating novel active biomaterials, either by embedding antimicrobial agents within hydrogels or by functionalizing the contact lens surface with antimicrobial components [18].

While, several studies have reported the synthesis of silver nanoparticles using plant extracts and their antimicrobial evaluation, there remains a limited number of studies that systematically incorporate biogenically synthesized AgNPs into contact lens-compatible hydrogels, and evaluate their antimicrobial efficacy alongside optical and biocompatibility criteria relevant to ophthalmic use.

Furthermore, although extracts such as oregano, eucalyptus, and willow have been employed by our group in prior work [11, 12], this study advances the field by: (i) Utilizing chamomile extract, which has unique flavonoid-rich phytochemistry, for the synthesis of AgNPs (**AgNPs(CHA)**), and(ii) Demonstrating superior antimicrobial performance of **AgNPs(CHA)** compared to previously reported AgNPs from other plant extracts (e.g., oregano or eucalyptus) in terms of MIC, MBC, and IZ values.

Additionally, to our knowledge, this is the first report of chamomile-derived AgNPs embedded in pHEMA hydrogels as functional materials for contact lenses that simultaneously exhibit: (i) potent antibacterial activity against major keratitis-causing pathogens (*P. aeruginosa*, *S. epidermidis*, and *S. aureus*), (ii) minimal cytotoxicity in HCEC cells under 24 h exposure, and (iii) in vivo biosafety in *Artemia salina* and *Allium cepa* models.

As part of our research on developing innovative antimicrobial materials [11, 12, 18–22] designed for use in contact lenses and their storage cases to minimize the risk of microbial infections, our team has adopted two distinct strategies. One strategy involves the use of biogenic silver nanoparticles synthesized with extracts from natural products, which serve as both reducing and capping agents [11, 12]. The second strategy involves utilizing small molecules, such as amino acids, metabolites, antimetabolites, or ingredients of natural products that are compatible with biological systems and also exhibit antimicrobial properties [11, 12, 18–22]. Our group recently reported the development of the materials pHEMA@AgNPs(ORLE)_2, pHEMA@AgNPs(ELE)_2, and pHEMA@AgNPs(WBE)_2, which incorporate silver nanoparticles—AgNPs(ORLE), AgNPs(ELE), and AgNPs(WBE)—synthesized using extracts from oregano leaves (ORLE), eucalyptus leaves (ELE), and willow bark (WBE), respectively [11, 12]. The antimicrobial activity of these materials against bacteria associated with microbial contamination of contact lenses and their storage cases highlights the potential of this approach and underscores the need for further research [11, 12]. Building on our ongoing research in this strategy, we report here the development of the silver hydrogel **pHEMA@AgNPs(CHA)**, which incorporates silver nanoparticles (**AgNPs(CHA)**) synthesized using chamomile extract. The materials were thoroughly characterized using RI, XRF, XRPD, TG–DTA, DTG/DSC, ATR-FTIR and UV–Vis spectroscopies. The antimicrobial potential of the materials was assessed against Gram-negative bacterial strains (*Pseudomonas aeruginosa* and *Escherichia coli*) and Gram-positive strains (*Staphylococcus epidermidis* and *Staphylococcus aureus*).

Therefore, the objectives of this study are:(i) to synthesize silver nanoparticles using chamomile extract and characterize them via UV–Vis, FTIR, and SEM; (ii) to incorporate the AgNPs into poly(2-hydroxyethyl methacrylate) (pHEMA) hydrogels suitable for ophthalmic use; (iii) to evaluate the antimicrobial activity of the AgNPs and the hydrogel-embedded AgNPs against *P. aeruginosa*, *E. coli*, *S. aureus*, and *S. epidermidis*, through disk diffusion (IZ), MIC, and MBC assays; (iv) to assess the cytotoxicity of the AgNPs in human corneal epithelial cells (HCECs) via MTT assay; and (v) to evaluate the in vivo biosafety of the hydrogels in *Artemia salina* and *Allium cepa* models.

## Results and discussion

### General aspects

**Scheme 2:**
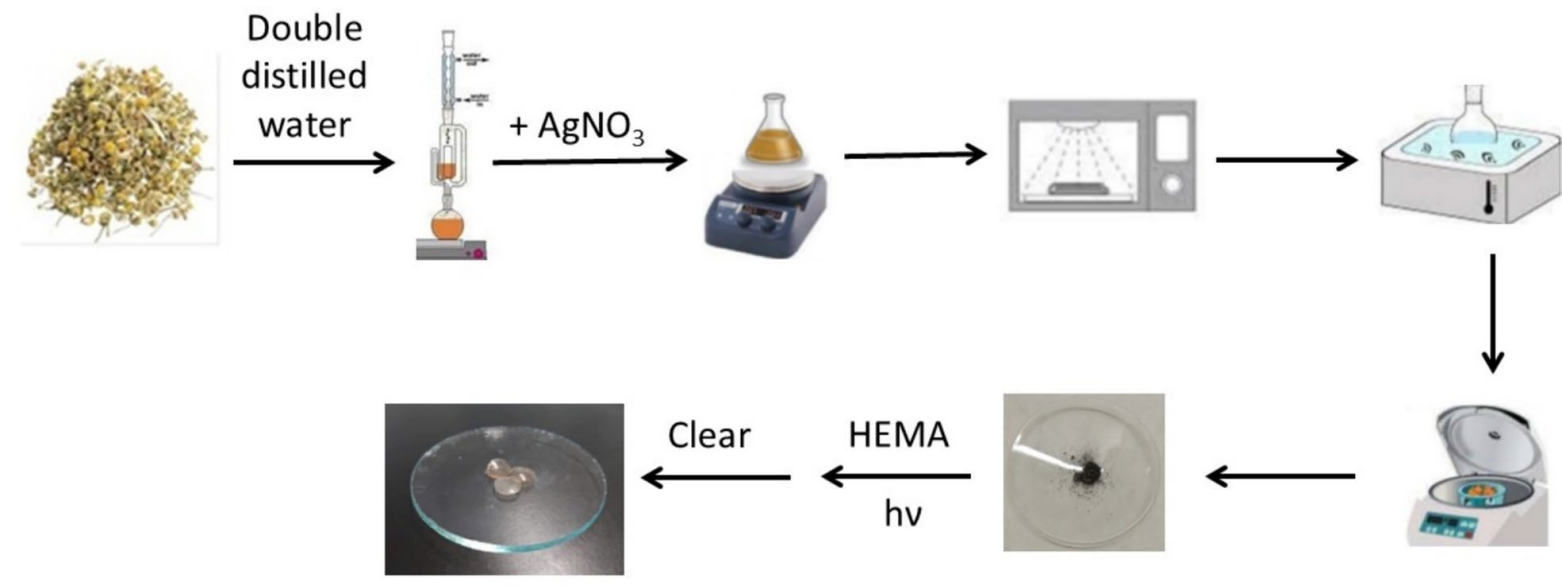
Formation mechanism of **AgNPs(CHA)**

Poly(2-hydroxyethyl methacrylate) (pHEMA), cross-linked with ethylene glycol dimethacrylate (EGDMA), forms the base material for a wide range of soft contact lenses intended for daily use [11, 12, 21–24]. To evaluate the antimicrobial efficacy of the contact lens material, **AgNPs(CHA)** nanoparticles (Scheme 2) were synthesized and incorporated into the pHEMA matrix during the polymerization process at concentrations of 1 mg/mL (**pHEMA@AgNPs(CHA)_1**) and 2 mg/mL (**pHEMA@AgNPs(CHA)_2**) [11, 12, 21–24] (Scheme 2). Discs of the material, 10 mm in diameter, were cut, thoroughly cleaned to eliminate any residual monomers, and subsequently stored either in sterilized 0.9% w/w NaCl solution or dried at 50 °C.

### Solid state

#### Refractive index

The refractive indices of the discs stored in saline solution were nearly identical, measuring 1.434 for pHEMA, 1.433 for **pHEMA@AgNPs(CHA)_1**, and 1.437 for **pHEMA@AgNPs(CHA)_2**. These values are consistent with those reported for similar materials, such as pHEMA@PtNPs (1.424–1.436) [11, 23], pHEMA@ORLE_2 (ORLE = oregano leaves extract) and pHEMA@AgNPs(ORLE)_2 (1.434 and 1.433, respectively), and pHEMA@AGMNA-1 (1.436) [11, 24]. Additionally, the refractive indices of other materials were determined as follows: pHEMA@AgNPs(WBE)_2 = 1.436 (WBE = willow bark extract), pHEMA@AGGLY-2 = 1.433 (AGGLY = [Ag₃(Gly)₂NO₃]ₙ, Gly = glycine), pHEMA@AGU-2 = 1.434 (AGU = [Ag(U)NO₃]ₙ, U = urea), pHEMA@AGSAL-2 = 1.437 (AGSAL = [Ag(salH)]₂, salH = salicylic acid). While ideal hydrogels typically exhibit refractive indices in the range of 1.372–1.381 [11, 24], the fabricated hydrogel demonstrates excellent transparency, with refractive indices ranging from 1.42 to 1.45 across the spectral range of 400 nm to 800 nm [11, 25, 26].

### X-ray fluorescence spectroscopy

**Fig. 1:**
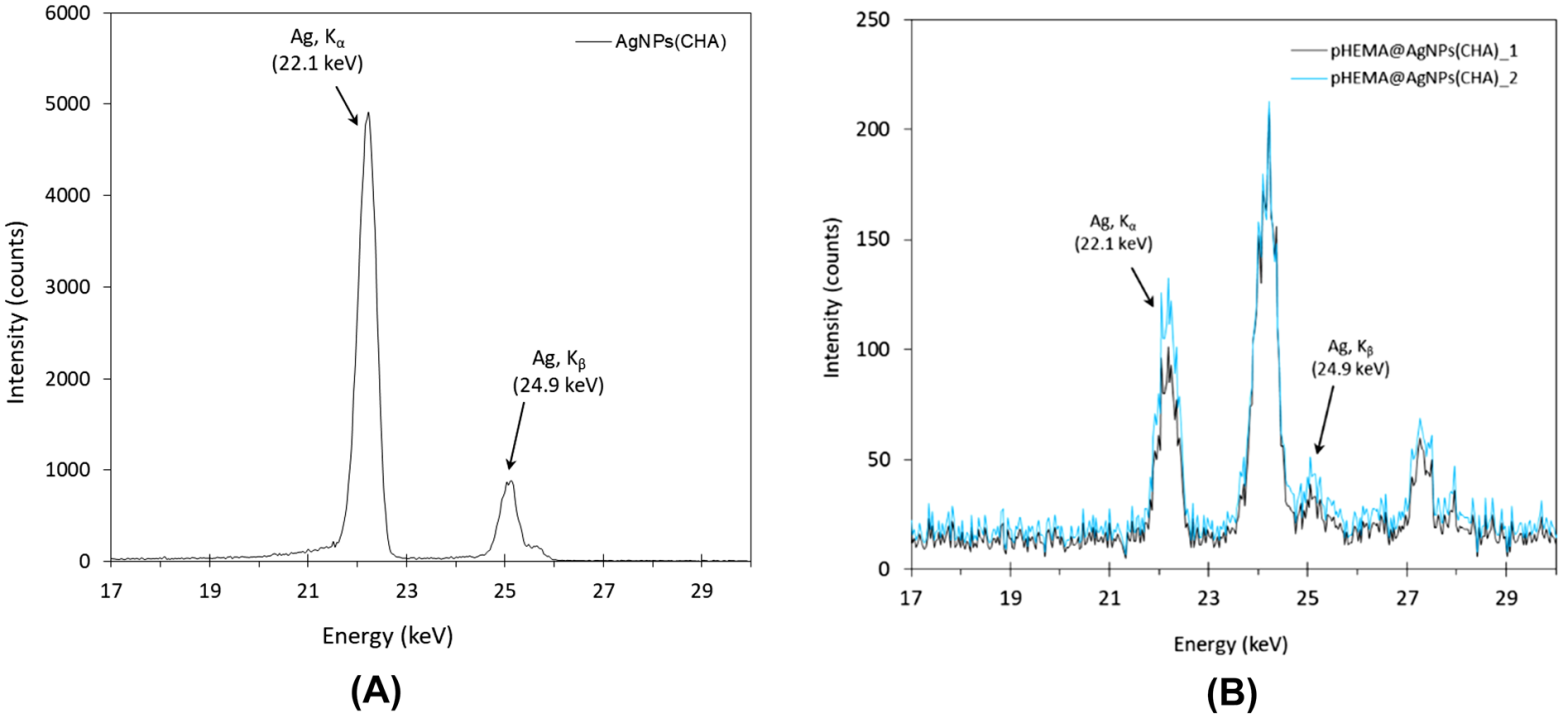
XRF spectra of the **AgNPs(CHA)** (**A**) nanoparticles and their hydrogels **pHEMA@AgNPs(CHA)_1** or **pHEMA@AgNPs(CHA)_2** (**B**). The Ag Ka emission light was used to determine Ag content within the samples

Dry **AgNPs(CHA)** and discs of **pHEMA@AgNPs(CHA)_1**) or **pHEMA@AgNPs(CHA)_2** were ground into powder. The elemental composition of the **AgNPs(CHA)** was analyzed using X-ray fluorescence (XRF) spectroscopy (Fig. 1A). The analysis revealed that silver constitutes 49% w/w. The elemental analysis of the **pHEMA@AgNPs(CHA)** materials was performed, revealing the presence of silver, attributed to the incorporation of silver nanoparticles (**AgNPs(CHA)**) into the pHEMA hydrogel matrix (Fig. 1B). For the **pHEMA@AgNPs(CHA)_1** and **pHEMA@AgNPs(CHA)_2** hydrogels, the silver content was found to be 0.15, and 0.19% w/w respectively.

### X-ray powder diffraction analysis (XRPD)

**Fig. 2:**
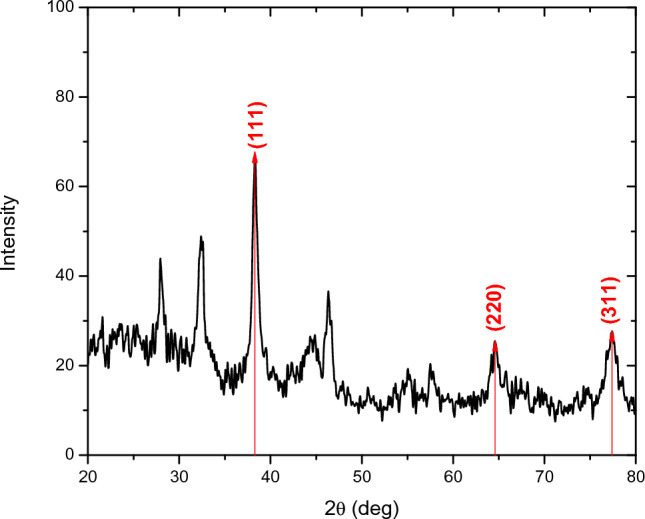
Powder X-Ray Diffraction of **AgNPs(CHA)**

The crystalline structure of silver nanoparticles (**AgNPs(CHA)**) was analyzed using powder X-ray diffraction (XRPD) (Fig. 2).

The diffractogram exhibited peaks at 2θ values of 38.30°, 44.81°, 64.52°, and 77.45°, corresponding to the (111), (200), (220), and (311) planes of silver, respectively, as identified from database references [27]. This indicates that the **AgNPs(CHA)** possess a face-centered cubic (FCC) crystal structure. Additionally, three peaks at 2θ values of 32.33°, 54.50° and 57.57° were observed, which may correspond to silver oxide (Ag₂O) nanoparticles.

The average crystallite size (D) of the **AgNPs(CHA)** was calculated using the Debye–Scherrer equation:

$${\text{D}} = 0.{9} \times \lambda /(\beta \times {\text{cos}}\theta )$$

where: λ is the X-ray wavelength, β is the full width at half maximum (FWHM) of the peak, determined using Gaussian fitting in Origin software, θ is the Bragg angle.

Calculations for the diffraction signals at 2θ values of 38.30, 64.52 and 77.45° are presented in Table 1. The Debye–Scherrer equation yielded an average crystallite size of 8.02 nm for the **AgNPs(CHA).**

**Table 1:** Calculation of silver nanoparticle crystallite size

2θ	FWHM	θ	θ radian	FWHM radian	Crystalline size D (nm)	Average size (nm)
38.3	0.79	19.15	0.33	0.014	9.5	8.02
64.52	0.78	32.26	0.56	0.014	8.6	
77.45	1.04	38.7	0.67	0.018	5.95	

Figure S1 presents the diffraction patterns of pHEMA, **pHEMA@AgNPs(CHA)_1**, **pHEMA@AgNPs(CHA)_2**, and the corresponding **AgNPs(CHA)** for comparison. The amorphous nature of pHEMA is also evident in the **pHEMA@AgNPs(CHA)_1** and **pHEMA@AgNPs(CHA)_2** materials. Due to the small concentration of **AgNPs(CHA)** in the composite, no crystalline phase peaks corresponding to **AgNPs(CHA)** are observed. As a result, the **pHEMA@AgNPs(CHA)_1** and **_2** materials remain amorphous, with the primary difference from the pHEMA diffraction pattern being the increased intensity of the shared peaks.

### Thermogravimetric analysis

#### Differential scanning calorimetry (DSC)

**Fig. 3:**
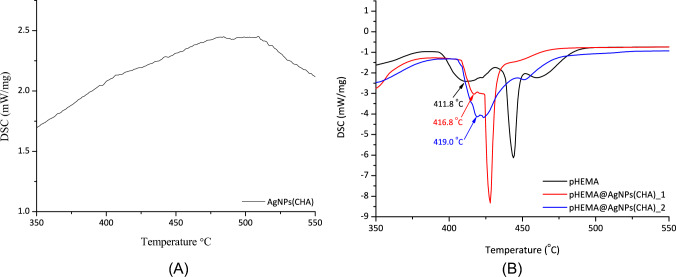
Differential Scanning Calorimetry (DSC) graphs of the powders of **AgNPs(CHA)** (**A**) and the corresponding of pHEMA, **pHEMA@AgNPs(CHA)_1** and **pHEMA@AgNPs(CHA)_2** (**B**)

To determine whether **AgNPs(CHA)** nanoparticles interact with pHEMA in the solid state, forming either a mixture or a composite material, DSC studies were conducted on the dry pHEMA powder as well as **pHEMA@AgNPs(CHA)_1** and **pHEMA@AgNPs(CHA)_2** (Fig. 3A, B). The endothermic transitions of the DSC diagram of the free pHEMA at 411.8 °C is observed at 416.8 °C **pHEMA@AgNPs(CHA)_1** and at 419.0 °C in **pHEMA@AgNPs(CHA)_2**, respectively, implying an interaction between the material’s components with pHEMA and therefore the formation of composite materials.

#### Thermal decomposition

**Fig. 4:**
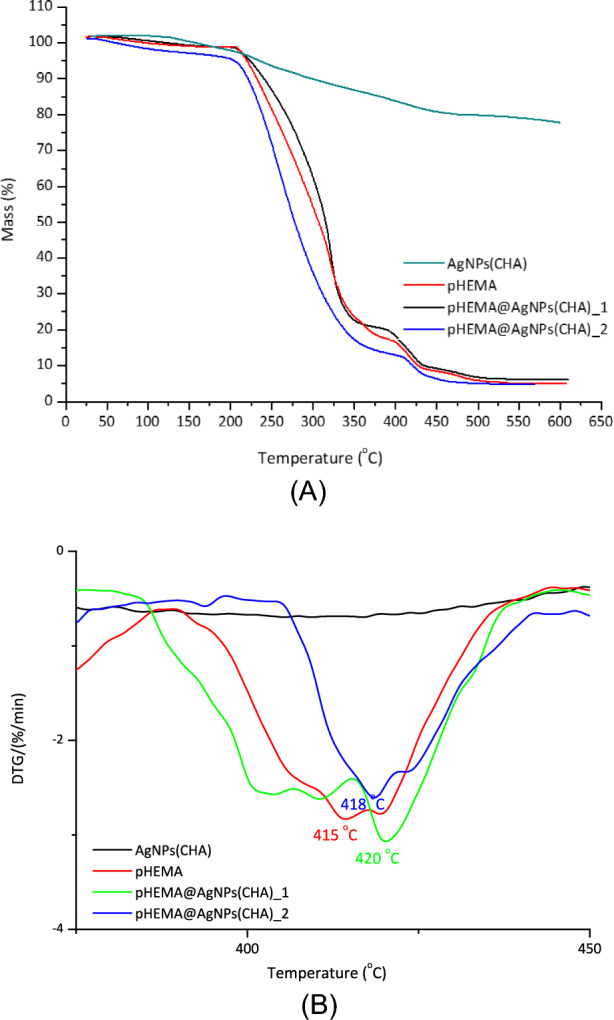
TG (**A**) and DTA (**B**) thermograms of **AgNPs(CHA)**, pure pHEMA and hydrogel composites **pHEMA@AgNPs(CHA)_1** and **pHEMA@AgNPs(CHA)_2**

TG/DTA analysis was conducted under air on the powdered samples of dry **AgNPs(CHA), pHEMA@AgNPs(CHA)_1**, and **pHEMA@AgNPs(CHA)_2**, with the temperature increasing at a rate of 10 °C min^−1^ from ambient up to 500 °C (Fig. S1). Figure 4A compares the thermographs of the composite materials **pHEMA@AgNPs(CHA)_1** and **pHEMA@AgNPs(CHA)_2**, alongside their individual components, **AgNPs(CHA)** and **pHEMA.** It is clear that the thermal response of the hydrogels **pHEMA@AgNPs(CHA)_1** and **pHEMA@AgNPs(CHA)_2** to increasing heat differs from that of their individual components, **AgNPs(CHA)** and pHEMA, providing strong evidence for the successful formation of the hydrogel-nanoparticle composite materials. Thus, despite that both the polymer pHEMA and the hydrogel composite **pHEMA@AgNPs(CHA)_1** and **pHEMA@AgNPs(CHA)_2** display a multi-step thermal degradation process, indicating that the main degradation mechanisms of the polymer matrix remain largely unaltered the Thermographs are not identical. Moreover, the primary decomposition stage associated with the polymer backbone occurs at a similar temperature range for both the pure polymer pHEMA and the composite materials **pHEMA@AgNPs(CHA)_1** and **pHEMA@AgNPs(CHA)_2**, showing that the core properties of the polymer are retained.

Figure 4B depicts the DTA diagrams of the **pHEMA@AgNPs(CHA)_1** and **pHEMA@AgNPs(CHA)_2**, and their ingredients **AgNPs(CHA)**, and pHEMA. The DTA diagrams show a shift in the decomposition temperature of pHEMA to higher values (415 °C (pHEMA), 418 and 420 C for **pHEMA@AgNPs(CHA)_1** and **_2**) indicating enhanced thermal stability. New thermal events, absent in the pure components, appear in the composite curve, suggesting interactions between the polymer and nanoparticles. These findings, if present in the composite DTA curve, validate the successful formation of the pHEMA**-AgNP** composite material with improved thermal properties and stability.

### ATR-FT-IR spectroscopy

**Fig. 5:**
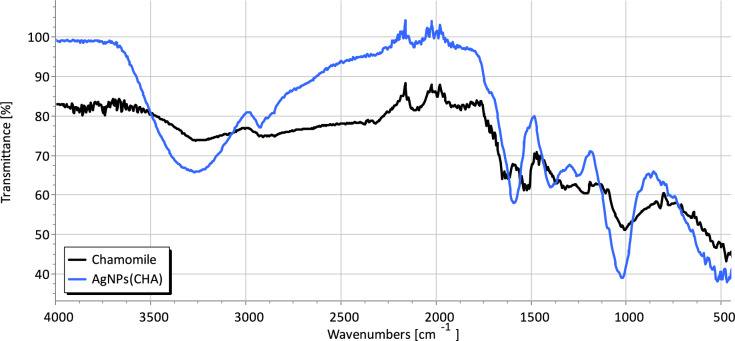
ATR-FTIR spectra of **AgNPs(CHA)** and CHA

Figure 5 presents the ATR-FT-IR spectra of CHA and its silver nanoparticles (**AgNPs(CHA**)). In the spectrum of CHA, the vibrational bands observed at 3273, 1636, 1625, 1320 1214 and 1005 cm⁻^1^ are attributed to its active functional groups, such as phenolic compounds, flavonoids, and terpenes. These groups, which are responsible for stabilizing the silver nanoparticles, are also identified in the spectrum of **AgNPs(CHA)** through common bands at 3273, 1589, 1388, 1252 and 1014 cm⁻^1^. The ATR-FT-IR spectra of **pHEMA@AgNPs(CHA)_1**, and **pHEMA@AgNPs(CHA)_2** are identical to the corresponding one of pHEMA as expected due to the very low contain of the **AgNPs(CHA)** in the pHEMA matrix (Fig. S3).

### Solid state UV–Vis

The presence of **AgNPs(CHA)** within the hydrogel matrix was confirmed using UV–Vis spectroscopy in the solid state. Solid-state UV–Vis spectroscopy was utilized because the pHEMA hydrogel is insoluble. Spectra were recorded for pHEMA, **AgNPs(CHA)**, **pHEMA@AgNPs(CHA)_1**, and **pHEMA@AgNPs(CHA)_2**. In the solid-state UV–Vis spectra, reflectivity was measured as a function of wavelength. To convert reflectivity into absorption, the Kubelka–Munk equation was applied:

$$A = \frac{{\left( {1 - \% R/100} \right)^{2} }}{2 \times \% R/100}$$

where A is the absorption and R% is the reflectivity.

**Fig. 6:**
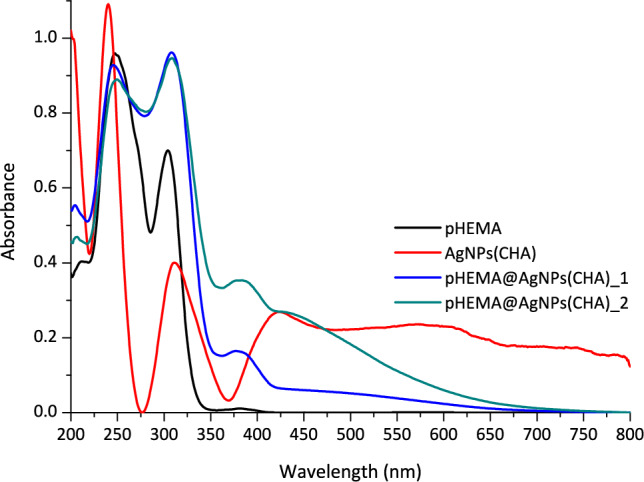
Solid state Uv–Vis of **AgNPs(CHA)**, pHEMA, **pHEMA@AgNPs(CHA)_1** and **pHEMA@AgNPs(CHA)_2**

The spectra indicate that for the materials **pHEMA@AgNPs(CHA)_1** and **pHEMA@AgNPs(CHA)_2**, a distinct band appears at 380 nm that is absent in the spectrum of both pHEMA (Fig. 6). This feature is attributed to the presence of **AgNPs(CHA)** and their characteristic surface plasmon resonance, observed in the range of 424 nm.

### Scanning electron microscopy (SEM)

The surface and morphology of the synthesized silver nanoparticles (**AgNPs(CHA)**) derived from chamomile extract were examined using scanning electron microscopy (SEM). In the image (Fig. S4), nanoparticles of varying sizes can be observed. Based on the analysis of the image at a magnification of 5 μm using ImageJ software, the average particle diameter was estimated to be 195 ± 15 nm.

### Solution studies

#### Ultraviolet–Visible spectroscopy (UV–Vis)

**Fig. 7:**
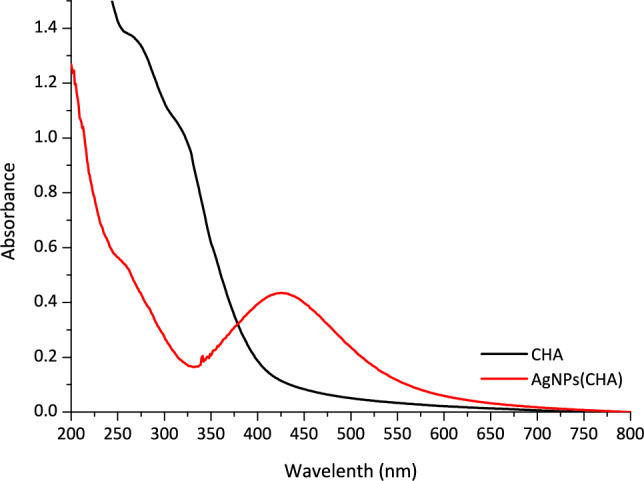
UV–Vis spectra of **AgNPs(CHA)** and chamomile in double-distilled (dd) water (1 mgr/mL)

UV–Vis spectroscopy was utilized to estimate the size of the silver nanoparticles. The UV–Vis spectrum of **AgNPs(CHA)** in a deionized water solution (1 mg/mL) displayed an absorbance maximum at λ_max_ = 424 nm (Fig. 7). This band corresponds to the Surface Plasmon Resonance (SPR), a phenomenon resulting from the collective oscillation of electrons on the nanoparticle surface [26, 28]. Since the size of nanoparticles is directly related to the λmax of the SPR, the diameter of **AgNPs(CHA)** was estimated to be approximately 37 nm [11]. This size is larger than the crystallite size determined from XRPD patterns, as expected.

#### Dynamic light scattering

The nanoparticles’ size distribution in solution was determined by dynamic light scattering (DLS) analysis. The average hydrodynamic diameter, in double distilled water was measured 351 nm (Fig. S5).

The observed discrepancy among the particle sizes determined by different techniques can be attributed to the inherent principles and limitations of each method. The crystallite size of 8.02 nm calculated from XRPD reflects the size of coherent diffracting domains, which may represent primary crystalline cores rather than whole particles. In contrast, the surface plasmon resonance (SPR) estimation (~ 37 nm) corresponds to the optical response of metallic nanoparticles in colloidal suspension and typically reflects the smaller population of well-dispersed particles. SEM image analysis revealed a larger average particle diameter of 195 ± 15 nm, which likely represents aggregated or agglomerated nanoparticles on the substrate surface during drying. Furthermore, dynamic light scattering (DLS) analysis in double-distilled water revealed an average hydrodynamic diameter of 351 nm (Fig. S5), which includes not only the metallic core but also the surrounding solvation layer and any capping agents from the green synthesis process. This value also reflects the presence of particle aggregates in solution. Taken together, these differences highlight the expected variability between techniques measuring either primary crystallites, individual particles, or aggregates in different physical states (solid vs. solution).

### Antibacterial activity of AgNPs(CHA)

#### Minimum inhibitory (MIC) and minimum bactericidal (MBC) concentrations

**Fig. 8:**
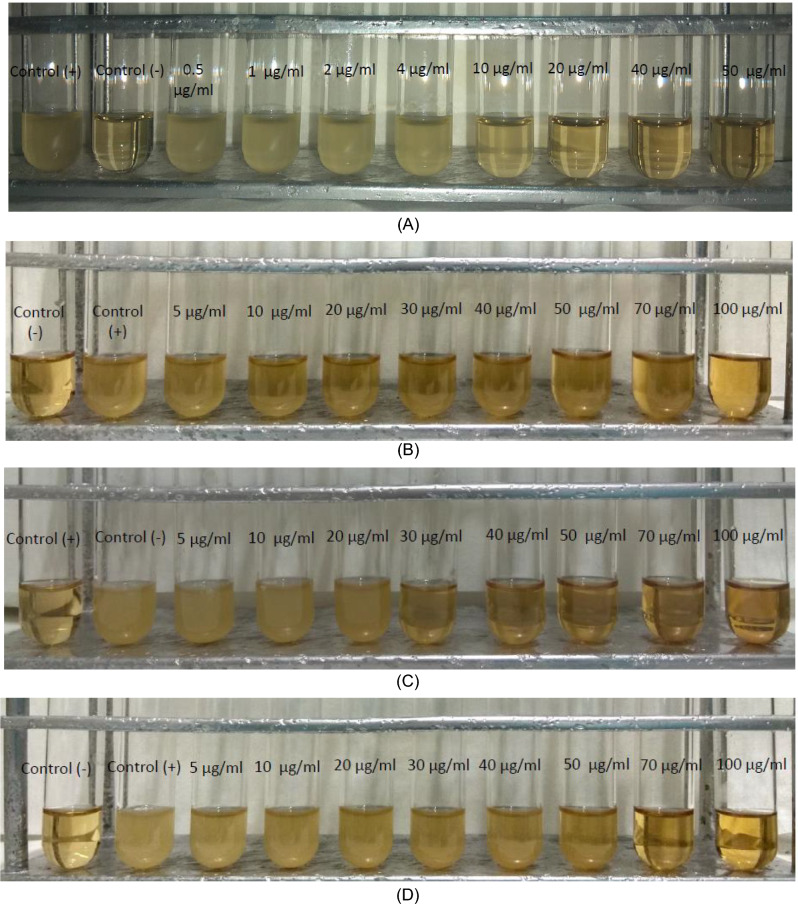
Broth culture of *P. aeruginosa* (**A**), *E. coli* (**B**), *S. epidermidis* (**C**) and *S. aureus* (**D**) in the presence of increasing concentrations of **AgNPs(CHA)**

Gram-negative bacteria (*P. aeruginosa* and *E. coli*) and Gram-positive bacteria (*S. epidermidis* and *S. aureus*) were selected for this study due to their prominence as pathogens in infections associated with contact lens use [11, 12, 18–22]. The antimicrobial activity of **AgNPs(CHA)** against these bacterial strains was assessed using the minimum inhibitory concentration (MIC), defined as the lowest concentration required to inhibit bacterial growth after a 20-h incubation period with the agent (Table 2, Fig. 8) [11, 12, 18–22].

**Table 2:** IZs, MICs and MBCs chamomile, **AgNPs(CHA)**, pHEMA, **pHEMA@AgNPs(CHA)_1** and **pHEMA@AgNPs(CHA)_2** and other silver nanoparticles against *P. aeruginosa, E. coli, S. epidermidis* and *S. aureus*

	*P. aeruginosa*	*E. coli*	*S. epidermidis*	*S. aureus*	References
MIC (μg/mL)					
**AgNPs(CHA)**	40.7 ± 7.4	> 100	48.6 ± 5.5	69.5 ± 7.6	*
**AgNPs(ORLE)**	139.5 ± 17.4	124.3 ± 12.9	272.2 ± 14.1	> 300	[11]
MBC (μg/mL)					
**AgNPs(CHA)**	40.0 ± 7.2	> 100	110 ± 11.3	85.0 ± 17.0	*
**AgNPs(ORLE)**	135.7 ± 35.2	> 300	> 300	> 300	[11]
MBC/MIC					
**AgNPs(CHA)**	1.0	–	2.3	1.2	*
**AgNPs(ORLE)**	1.0	–	–	–	[1]
IZ (mm)					
**AgNPs(CHA) 1 mg/mL**	16.0 ± 0.6	11.1 ± 0.9	15.8 ± 1.2	15.1 ± 0.2	*
Chamomile 1 mg/mL	ND	ND	ND	ND	*
**AgNPs(CHA) 2 mg/mL**	17.5 ± 0.4	12.4 ± 0.8	17.0 ± 0.0	16.9 ± 1.1	*
Chamomile 2 mg/mL	ND	ND	ND	ND	*
**AgNPs(ORLE) 2 mg/mL**	13.1 ± 1.6	12.3 ± 0.7	12.7 ± 1.7	14.8 ± 1.1	[1]
**AgNPs(ELE)**	15.2 ± 1.2		17.0 ± 1.1	15.2 ± 1.6	[12]
**AgNPs(WBE)**	11.9 ± 0.9		–	11.0 ± 0.0	[12]
**pHEMA**	ND	ND	–	ND	*
**pHEMA@AgNPs(CHA)_1**	10.8 ± 0.5	ND	11.0 ± 0.9	11.0 ± 0.0	*
**pHEMA@AgNPs(CHA)_2**	11.3 ± 0.7	ND	10.8 ± 1.5	15.0 ± 1.9	*
**pHEMA@AgNPs(ORLE)_2**	10.3 ± 0.7	ND	11.0 ± 1.9	10.3 ± 0.7	[11]
**pHEMA@AgNPs(ELE)_2**	12.3 ± 1.7	-	13.2 ± 1.2	13.2 ± 1.4	[12]
**pHEMA@AgNPs(WBE)_2**	11.2 ± 0.3	-	11.0 ± 1.0	11.5 ± 0.6	[12]
Bacteria viability (%)					
**pHEMA@AgNPs(CHA)_1**	0.9 ± 0.2	100.0	1.9 ± 1.0	4.4 ± 1.5	*
**pHEMA@AgNPs(CHA)_2**	0.8 ± 0.2	100.0	3.0 ± 1.5	7.6 ± 2.0	*
**pHEMA@AgNPs(ORLE)_2**	66.5	88.3	77.7	59.6	[11]
**pHEMA@AgNPs(ELE)_2**	28.3	–	6.8	85.3	[12]
**pHEMA@AgNPs(WBE)_2**	73.6	–	81.6	82.5	[12]

*This study; ND = No inhibitory zone (IZ) was developed; ORLE = oregano leaves extract; ELE = Eucalyptus leaves, WBE = willow bark extract

The MIC values of **AgNPs(CHA)** were determined to be 40.7, > 100, 48.6, and 69.5 μg/mL for *P. aeruginosa*, *E. coli*, *S. epidermidis*, and *S. aureus*, respectively.

**Fig. 9:**
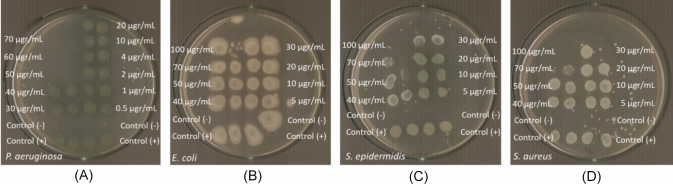
MBC of **AgNPs(CHA)** against *P. aeruginosa* (**A**)*, E. coli* (**B**)*, S. epidermidis* (**C**) and *S. aureus* (**D**)

The minimum bactericidal concentration (MBC) is defined as the lowest concentration of an antibacterial agent required to eliminate 99.9% of the bacterial inoculum [11, 12, 18–22]. The MBC values of the tested agents are presented in Table 2 and Fig. 9. For **AgNPs(CHA)**, the MBC values were determined to be 40.0, > 100, 110.0, and 85.0 μg/mL against *P. aeruginosa*, *E. coli*, *S. epidermidis*, and *S. aureus*, respectively.

The MBC/MIC ratio is a key parameter used to distinguish between bactericidal and bacteriostatic activity of an antimicrobial agent. When the MBC/MIC ratio is ≤ 2, the agent is classified as bactericidal. Conversely, a MBC/MIC ratio ≥ 4 indicates bacteriostatic activity, where bacterial growth is inhibited without significant killing.

Based on the MBC/MIC ratios (1–2.3), **AgNPs(CHA)** is classified as a bactericidal agent against *P. aeruginosa*, *S. epidermidis*, and *S. aureus* (MBC/MIC ratio ≤ 2).

#### Viability of microbes upon their incubation with materials

**Fig. 10:**
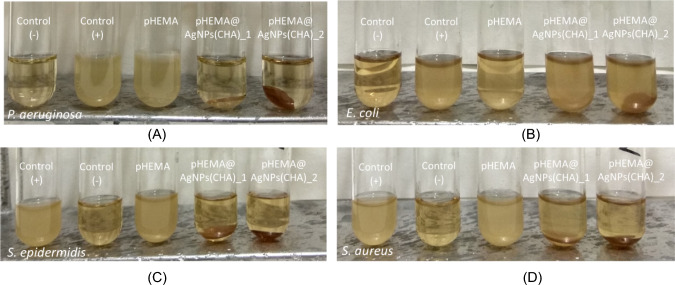
Bacterial [*P. aeruginosa* (**A**), *E. coli* (**B**), *S. epidermidis* (**C**) and *S. aureus* (**D**)] viability when they are incubated over pHEMA, **pHEMA@AgNPs(CHA)_1**, and **pHEMA@AgNPs(CHA)_2** discs under continuous stirring

Given the antimicrobial efficacy of **AgNPs(CHA),** (see above) the nanoparticles were incorporated into the hydroxyethyl methacrylate (pHEMA) matrix at a concentration of 1 mg/mL (**pHEMA@AgNPs(CHA)_1**) or 2 mg/mL (**pHEMA@AgNPs(CHA)_2**) during the polymerization process. The materials (**pHEMA@AgNPs(CHA)_1** or **pHEMA@AgNPs(CHA)_2**) were cut in to 10 mm diameter discs. These discs were placed in tubes containing Gram-negative (*P. aeruginosa* and *E. coli*) and Gram-positive (*S. epidermidis* and *S. aureus*) bacterial strains (Fig. 10).

**Fig. 11:**
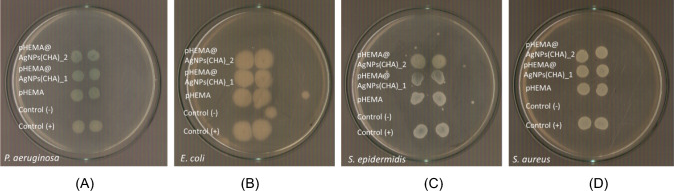
Bacterial colonies of *P. aeruginosa* (**A**), *E. coli* (**B**), *S. epidermidis* (**C**) and *S. aureus* (**D**) grown in agar plates when 4 μL of the supernatants of the solutions were used, which were initially treated with discs of pHEMA and **pHEMA@AgNPs(CHA)_1**, and **pHEMA@AgNPs(CHA)_2**

The calculated bacterial viability percentages of *P. aeruginosa, S. epidermidis*, and *S. aureus* after incubation with **pHEMA@AgNPs(CHA)_1** and **pHEMA@AgNPs(CHA)_2** were remarkably low, ranging from 0.9% to 6.8% and 0.8% to 8.3%, respectively (Table 2, Fig. 10). Neither the **pHEMA@AgNPs(CHA)_1** nor the **pHEMA@AgNPs(CHA)_2** discs showed effectiveness against *E. coli*, consistent with the lack of activity of **AgNPs(CHA)** themselves against *E. coli*. No effect on bacterial viability was observed when treated with pure pHEMA discs. In a subsequent experiment, 4 μL of bacterial broth solution, previously incubated for 20 h with pHEMA, **pHEMA@AgNPs(CHA)_1**, or **pHEMA@AgNPs(CHA)_2** discs, was used to grow colonies on agar plates (Fig. 11). The results showed that the materials effectively inhibit microbial growth only when they are directly present within the microbial cultures.

#### Determination of the inhibition Zone (IZ) through agar disk-diffusion method

**Fig. 12:**
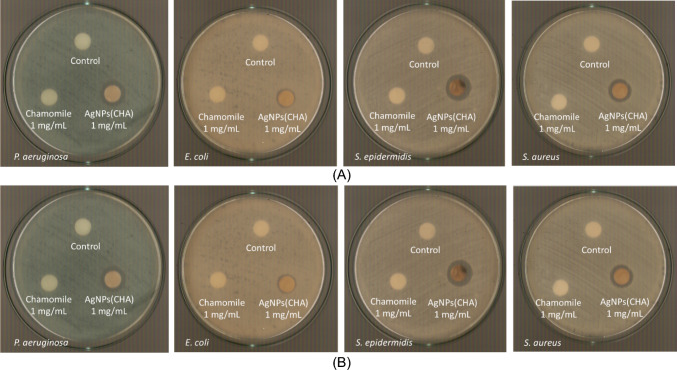
IZs which are developed in agar plates of *P. aeruginosa*, *E. coli, S. epidermidis and S. aureus* by chamomile and **AgNPs(CHA)** at 1 mg/mL (**A**) and 2 mgr/mL (**B**)

**Fig. 13:**
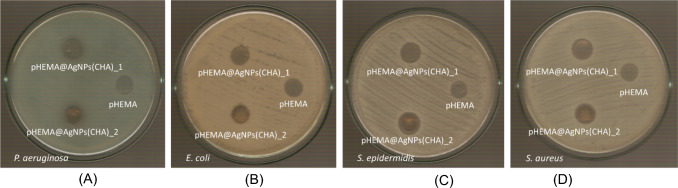
Inhibition zones on *P. aeruginosa* (**A**)*, E. coli* (**B**)*, S. epidermidis* (**C**) and *S. aureus* (**D**) bacteria developed by pHEMA, **pHEMA@AgNPs(CHA)_1**, and **pHEMA@AgNPs(CHA)_2** discs

The highly promising results of the agents against bacteria associated with microbial keratitis led to the use of the agar disk diffusion method. This approach was employed to confirm the effectiveness of the new agents in eliminating the bacteria and to evaluate the microorganisms' sensitivity in the presence of these materials [8, 9, 15–19]. Table 2 summarizes the inhibition zones developed for CHA and **AgNPs(CHA)** at concentrations of 1 mg/mL and 2 mg/mL, as well as for **pHEMA**, **pHEMA@AgNPs(CHA)_1,** and **pHEMA@AgNPs(CHA)_2** discs, against Gram-negative (*P. aeruginosa* and *E. coli*) and Gram-positive (*S. epidermidis* and *S. aureus*) bacteria (Figs. 12, 13).

The bacteria exhibit low sensitivity to **AgNPs(CHA),** with inhibition zone (IZ) diameters ranging from 11.1 to 16.0 mm at a concentration of 1 mg/mL and 12.4–17.0 mm at 2 mg/mL (Fig. 12). The chamomile extract (CHA) shows no antimicrobial activity at either concentration of 1 mg/mL or 2 mg/mL (Fig. 12). Based on the diameter of the inhibition zone (IZ), microbial strains are classified into three categories in response to an antimicrobial agent: (i) strains with an IZ diameter ≥ 17 mm are considered susceptible, (ii) strains with an IZ diameter between 13 and 16 mm (13 ≤ IZ ≤ 16 mm) are classified as intermediate, and (iii) strains with an IZ diameter ≤ 12 mm are regarded as resistant [8, 9, 15–19]. Therefore, the bacterial strains *P. aeruginosa*, *S. epidermidis*, and *S. aureus* are classified as intermediate at a concentration of 1 mg/mL of **AgNPs(CHA)** and as susceptible at 2 mg/mL. In contrast, the *E. coli* strain is classified as resistant at both tested concentrations.

**Scheme 3:**
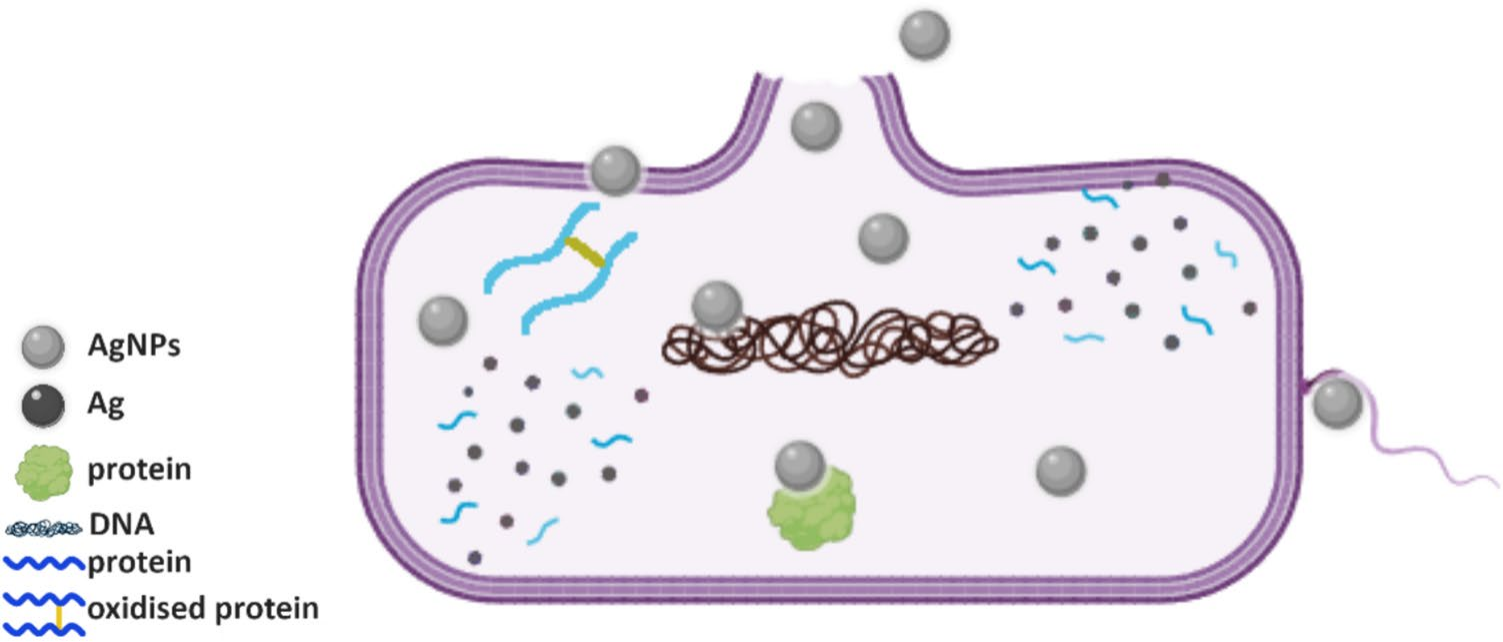
Proposed possible mechanisms of bacterial inactivation [5] by **AgNPs(CHA)** (Grey Spheres): (**i**) direct contact with the bacterial cell membrane, leading to loss of membrane integrity; (**ii**) interaction with bacterial DNA, inhibiting its replication; and (**iii**) disruption of membrane proteins amino acid site group oxidation (e.g. disulfide bond formation) accompanied by the release of free silver metal (Black Spheres) from the nanoparticles

The inhibition zones produced by **pHEMA@AgNPs(CHA)_1** and **pHEMA@AgNPs(CHA)_2** range from 10.8 to 11.0 mm and 10.8 to 15.0 mm, respectively, for the strains *P. aeruginosa*, *S. epidermidis*, and *S. aureus* (Table 2, Fig. 13). In contrast, *E. coli* shows no sensitivity to either material. No inhibition zones were observed for the pure pHEMA discs against any bacterial strain. The **pHEMA@AgNPs(CHA)_1** and **pHEMA@AgNPs(CHA)_2** discs, with a diameter of 10 mm, exhibit smaller inhibition zones compared to soaked paper discs treated with **AgNPs(CHA)** solutions (1 or 2 mg/mL), suggesting minimal or negligible release of the active ingredients from the discs. Scheme 3 illustrates the proposed possible mechanisms (i-iii) of action of AgNPs against bacteria [5].

#### In vitro* toxicity against normal human corneal epithelial Cells (HCECs)*

Human corneal epithelial cells (HCEC) are widely used to evaluate the toxicity of various ophthalmic drugs, pharmaceutical excipients, and natural tear substitutes [11, 12, 18–22, 29]. In this study, the in vitro toxicity of **AgNPs(CHA)** was assessed against HCEC cells after a 48-h incubation using the sulforhodamine B (SRB) assay. The IC_50_ value of **AgNPs(CHA)** was determined to be 20.9 ± 1.8 μg/mL. Silver nanoparticles (AgNPs) often display dose- and size-dependent toxicity, with reported IC_50_ values ranging broadly depending on surface coating, synthesis method, and target cell line. For example: IC_50_ values for AgNPs against HCEC’s, suggest low toxicity [30].

To further assess the cell viability in the presence of the materials **pHEMA@AgNPs(CHA)_1** and **pHEMA@AgNPs(CHA)_2**, the materials were incubated with HCEC cells for 24 h, matching the maximum incubation period of the materials with bacteria. The cell viability percentages were found to be 89.4 ± 9.4% and 62.0 ± 7.5%, respectively, compared to cells incubated with pure pHEMA. According to ISO 10993–5, if the percent of cell viability is higher than 70%, then the material should be considered as non-cytotoxic [11, 12, 18–22]. Thus, **pHEMA@AgNPs(CHA)_1** can be considered as non cytotoxic, since the cell viability percentage is higher than 70% [11, 12, 18–22, 31]. However, when the materials were incubated for 48 h, cell viability decreased to 32.8 ± 7.5% for **pHEMA@AgNPs(CHA)_1** and 18.8 ± 7.8% for **pHEMA@AgNPs(CHA)_2**.

#### In vivo toxicity against *Artemia salina*

The in vivo toxicity of the materials was also evaluated using the brine shrimp *Artemia salina* over a 24-h period. *Artemia salina*, a zooplanktonic crustacean, is commonly used as a model organism for acute toxicity screening of materials [11, 12, 18–22, 32]. No mortality was observed for either material, indicating their non-toxic behavior in this model system.

#### In vivo genotoxicity by *Allium cepa* test

The *Allium cepa* assay is a widely used method for evaluating the toxicity and genotoxicity of chemicals and potential drugs. The Environmental Protection Agency (EPA) and the United Nations Environment Program have standardized its use as a bioindicator. Both the World Health Organization (WHO) and the EPA acknowledge this bioassay as a reliable and effective tool for assessing potential genotoxicity in vivo [33, 34]. The *Allium cepa* assay has demonstrated a strong correlation with mammalian test systems [33]. It is a versatile tool for evaluating chromosomal irregularities, mitotic anomalies, and DNA damage caused by mutagens. This includes the assessment of mitotic anomalies, quantified by the mitotic index (%), and DNA damage, measured through chromosomal aberrations (%) [33].

**Fig. 14:**
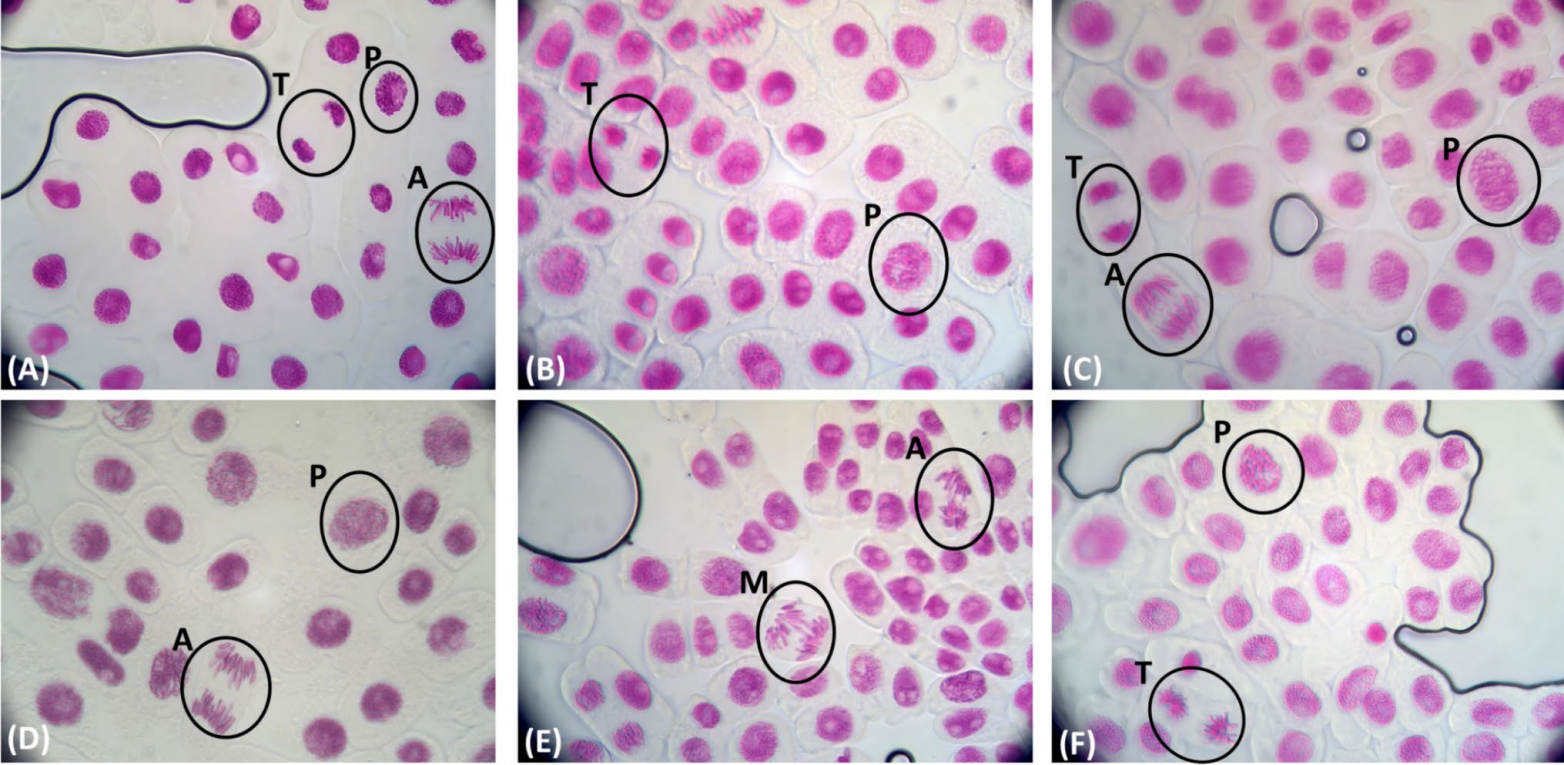
*Allium cepa* meristematic cells exposed to materials. Untreated Allium cepa meristematic cells (**A**) (P = prophase, A = anaphase, M = metaphase, T = telophase). Allium cepa meristematic cells exposed with **AgNPs(CHA)** 1 mg/mL (**B**), **AgNPs(CHA)** 2 mg/mL (**C**), pHEMA (**D**), **pHEMA@AgNPs(CHA)_1** (**E**) and **pHEMA@AgNPs(CHA)_2** (**F**)

Incubation of *Allium cepa* with **AgNPs(CHA)** at concentrations of 1 and 2 mg/mL for 48 h showed no effect on the mitotic index or chromosomal abnormalities. This indicates that these concentrations are neither mutagenic nor genotoxic in vivo, compared to untreated cells (Table 3, Fig. 14). A similar trend was observed with the materials **pHEMA@AgNPs(CHA)_1** and **pHEMA@AgNPs(CHA)_2**, further supporting the non-toxic behavior of these materials (Table 3, Fig. 14).

**Table 3:** The mitotic index % (MI) and the chromosomal aberrations % (CA) observed when *Allium cepa* was incubated with **AgNPs(CHA)** at 1 and 2 mg/mL, pHEMA, **pHEMA@AgNPs(CHA)_1** and **pHEMA@AgNPs(CHA)_2**

	MI	CA
Untreated cells	8.3 ± 0.4	0.0 ± 0.0
**AgNPs(CHA)** 1 mg/mL	6.6 ± 1.5	0.7 ± 0.4
**AgNPs(CHA)** 2 mg/mL	8.8 ± 1.1	1.3 ± 0.2
pHEMA	7.7 ± 1.0	0.3 ± 0.0
**pHEMA@AgNPs(CHA)_1**	9.2 ± 1.5	0.8 ± 0.3
**pHEMA@AgNPs(CHA)_2**	9.0 ± 1.2	0.0 ± 0.0

## Conclusion

The integration of **AgNPs(CHA)** nanoparticles into the pHEMA matrix resulted in the successful development of composite materials, **pHEMA@AgNPs(CHA)_1** and **pHEMA@AgNPs(CHA)_2**, which exhibit promising properties for potential applications in antimicrobial contact lenses. The refractive indices of the materials were comparable to those of standard pHEMA hydrogels, maintaining high transparency suitable for optical applications. XRF and XRPD analyses confirmed the successful incorporation of silver nanoparticles into the pHEMA matrix, with a consistent amorphous structure of the composites.

**Fig. 15:**
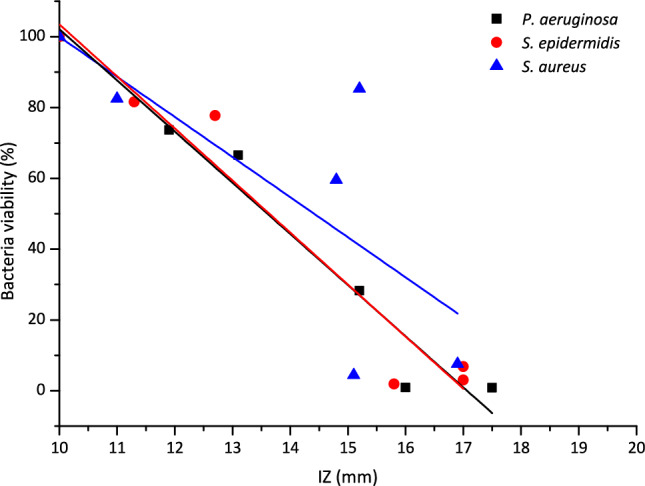
Inhibition zones (IZ’s) caused by **AgNPs(CHA)** vs % Bacterial viability treated with pHEMA@AgNPs against *P. aeruginosa*, *S. epidermidis* and *S. aureus*

The new materials (**pHEMA@AgNPs(CHA)_1** and **pHEMA@AgNPs(CHA)_2**) demonstrated significant antimicrobial efficacy against *P. aeruginosa*, *S. epidermidis*, and *S. aureus*, with low bacterial viability percentages and inhibition zones indicative of bactericidal activity. However, they were ineffective against *E. coli*, consistent with the intrinsic limitations of **AgNPs(CHA)** against this strain. In comparison, to other biogenic silver nanoparticles derived from oregano leaves (ORLE), eucalyptus leaves (ELE), and willow bark (WBE) extracts (Table 2), **AgNPs(CHA)** demonstrate superior effectiveness, exhibiting the lowest MIC values and the highest inhibition zones (IZ) against all tested bacterial strains. Specifically, the IZs of **AgNPs(CHA)** at 2 mg/mL range from 12.4 to 17.5 mm for *P. aeruginosa*, *E. coli*, *S. epidermidis*, and *S. aureus*. In contrast, the IZs for AgNPs(ORLE), AgNPs(ELE), and AgNPs(WBE) at the same concentration fall within 12.3–14.8 mm, 15.2–17.0 mm, and 11.0–11.9 mm, respectively. Additionally, the larger the IZ formed by AgNPs, the greater the bacterial elimination rate achieved by the hydrogels (pHEMA@AgNPs) (Fig. 15). Notably, none of the original plant extracts alone exhibited antibacterial activity (Table 2).

The materials were found to be non-cytotoxic under short-term (24-h) exposure to HCEC cells, with **pHEMA@AgNPs(CHA)_1** meeting ISO 10993–5 criteria for biocompatibility. However, prolonged exposure (48 h) led to a notable decrease in cell viability, highlighting the importance of limiting exposure duration. Similarly, the composites pHEMA@AgNPs(ORLE)_2, pHEMA@AgNPs(ELE) _2, and pHEMA@AgNPs(WBE)_2 exhibit comparable cell viability percentages when tested on HCEC cells. Furthermore, the brine shrimp (*Artemia salina*) and *Allium cepa* assays confirmed the non-toxic and non-genotoxic nature of **AgNPs(CHA)** and the associated composite materials at the tested concentrations.

These findings suggest that **pHEMA@AgNPs(CHA)_1** is promising candidate for antimicrobial applications, particularly in the development of contact lenses for preventing microbial keratitis. Further optimization *and *in vivo evaluations are warranted to ensure their safety and efficacy in practical use.

In general, and as evidence of the methodological innovation brought by this study: (i) this is the first report of synthesizing silver nanoparticles (AgNPs) using chamo-mile extract for subsequent in-corporation into pHEMA-based hydrogel matrices de-signed for ophthalmic applications. (ii) Unlike previous methods which often use thermal or chemical reduction, the AgNPs were synthesized here under ambient conditions, using chamomile extract as stabilizing agent—a green and eco-friendly route with no external energy input. (iii) The hydrogel formulation process was adapted to ensure uniform dispersion of **AgNPs(CHA)** and preserve their physicochemical integrity, while maintaining optical clarity and softness appropriate for contact lens applications. (iv) The multitier biosafety evaluation, including both in vitro cytotoxicity (HCEC) and in vivo biocompatibility in *Artemia salina* and *Allium cepa*, provides a comprehensive validation pipeline not typically integrated into previous AgNP-hydrogel studies.

## Experimental

### Materials and instruments

All solvents used were of reagent grade. DMSO was supplied by Riedel–de Haen (Seelze, Germany). Double distilled water for the experiments was freshly prepared and was obtained using the BIDY WATER BI-DISTILLER B.E. 115 apparatus manufactured by BICASA, MILANO, ITALY. Silver nitrate was purchase from Degussa (Berlin, Germany). 2-Hydroxyethyl methacrylate (pHEMA), ethylene glycol dimethacrylate (EGDMA, Merck), and diphenyl(2,4,6-trimethylbenzoyl)phosphine oxide (TPO 97%, Sigma Aldrich), along with sodium chloride (NaCl, Merck) and hydrochloric acid (HCl 37%, Merck), were used. Tryptone and soy peptone were purchased from Biolife, Milano, Italy. Additionally, tryptone (tryptophan medium), beef extract powder, bacteriological peptone, and soy peptone were also obtained from Biolife. Agar and yeast extract were acquired from Fluka Analytical. Sodium chloride, D(+)-glucose, and dipotassium hydrogen phosphate trihydrate were purchased from Merck. Dulbecco’s Modified Eagle’s Medium (DMEM), fetal bovine serum, glutamine, and trypsin were obtained from Gibco, Glasgow, UK. Phosphate-buffered saline (PBS) was purchased from Sigma-Aldrich. For the toxicity experiments, brine shrimp eggs (Artemia salina) were sourced from Ocean Nutrition.

Melting points were determined in open tubes using a Stuart Scientific apparatus and are uncorrected. Electronic absorption spectra were obtained using a UV-1600 PC series spectrophotometer from VWR International GmbH, Darmstadt, Germany. ATR-FTIR spectra, spanning the range of 4000–370 cm⁻^1^, were recorded with a Cary 670 FTIR spectrometer from Agilent Technologies. XRF measurements were performed using an Am–241 radioisotopic source (exciting radiation 59.5 keV) and a Rigaku NEX QC EDXRF analyzer (Austin, TX, USA). DTA/TG measurements were carried out using a DTG/TG NETZSCH STA 449C. For XRPD studies, a D8 Advance Bruker diffractometer with CuKα radiation (40 kV, 40 mA, λKα) and a monochromator system for the diffracted beam were used.

#### Preparation of ***AgNPs(CHA)***

Dry chamomile leaves (4 g) were refluxed in 50 mL of deionized water using a Soxhlet apparatus for 4 h. The chamomile extract (CHA) solution was filtered off to give the chamomile leaves extract. In a 250 mL beaker, 10 mL of CHA and 170 mg of silver nitrate (AgNO₃, 1 mmol) were added. The beaker was then placed in a microwave oven (700 W) and it irradiated for 2–3 min 5-s intervals. Following this, the solution was sonicated for 10 min. The solution was centrifuged at 6000 rpm for 20 min, and the silver nanoparticles **AgNPs(CHA)** were collected.

**CHA**: yellow powder, ATR-IR (cm^−1^): 3260w, 2109 s, 1649 s, 1541 s, 1316 m, 1219 m, 1009vs, 821 m, 474 m; UV–Vis (ddH_2_O): λ_max_ = 262 nm.

**AgNPs(CHA)**: content of Ag in **AgNPs(CHA)**: 49% w/w; IR (cm^−1^): 3273w, 2924 m, 1592vs, 1395 s, 1256 s, 1022vs, 520 m, 469 m; UV–Vis (ddH_2_O): λ_max_ = 424 nm.

#### Synthesis of pHEMA@AgNPs(CHA)_1 and pHEMA@AgNPs(CHA)_2

Hydrogels of silver nanoparticles (**AgNPs(CHA)**) dispersed in pHEMA were prepared as previously described [11, 12, 18–22]. Briefly, 2.7 mL of HEMA was mixed with 2 mL of double-distilled water (ddw), containing **AgNPs(CHA)** at concentrations of 1 and 2 mg/mL, and 10 mL of ethylene glycol dimethacrylate (EGDMA). The solution was degassed by bubbling nitrogen through it for 15 min. Trimethylbenzoyldiphenylphosphine oxide (TPO) initiator (6 mg) was then added, and the mixture was stirred for 5 min at 800 rpm. The solution was poured into a mold and exposed to a UV mercury lamp (λmax = 280 nm, 15 watts) for photopolymerization for 40 min. Unreacted monomers were removed by immersing the gel in water for 15 min. Discs with a diameter of 10 mm were cut and washed by immersion in water, 0.9% NaCl, and 0.1 M HCl, followed by another wash in water. The discs were then dried at 40 °C until no further weight change occurred.

***pHEMA@AgNPs(CHA)_1****:* Ag content in **pHEMA@AgNPs(CHA)_1**: 0.15% w/w IR (cm^−1^): 3407w, 2922 s, 2852 m, 1706vs, 1453 s, 139 m, 1246 s, 1150vs, 1071 s, 1025 s, 965 m, 945 m, 898 m, 844 m, 747 s, 515 m, 439 s, UV–Vis (solid state): λ_max_ = 380 nm.

***pHEMA@AgNPs(CHA)_2****:* Ag content in **pHEMA@AgNPs(CHA)_2**: 0.19% w/w IR (cm^−1^): 3407w, 2922 s, 2852 m, 1706vs, 1453 s, 139 m, 1246 s, 1150vs, 1071 s, 1025 s, 965 m, 945 m, 898 m, 844 m, 747 s, 515 m, 439 s, UV–Vis (solid state): λ_max_ = 380 nm.

#### Refractive indexes

The refractive indices of the lenses were measured using an Abbe refractometer (NAR–1 T, Atago Co., Ltd., Tokyo, Japan) at 24 °C.

#### X-ray powder diffraction (XRPD)

The samples were analyzed using X-ray powder diffraction with a D8 Advance Bruker diffractometer, utilizing CuKα radiation (40 kV, 40 mA, λKα) and a monochromator system for the diffracted beam. X-ray powder diffraction patterns were recorded in the 2θ range of 2° to 80°, with a step size of 0.02° and a time interval of 2 s per step. All samples analyzed with the diffraction meter were in fine-grained powder form.

#### Thermogravimetric differential thermal analysis (TG–DTA), differential scanning calorimetry (DTG/DSC)

DTA/TG measurements were performed using a DTG/TG NETZSCH STA 449C. For these measurements, the samples were placed in a platinum capsule on one side of the thermal scale, while α-alumina was used as the reference on the opposite side. The temperature was increased at a rate of 10 °C/min over a range of 25–500 °C, and the measurements were conducted in air. All samples analyzed were in fine-grained powder form.

### Biological tests

#### Bacterial strains

For the antibacterial experiments, the strains *S. epidermidis* (ATCC® 14,990™), *S. aureus subsp. aureus* (ATCC® 25923™), *P. aeruginosa* and *E. coli* were used. The Gram negative bacterial strains *P. aeruginosa* and *E. coli* were kindly donated by the Laboratory of Biochemistry at the University of Ioannina, Greece.

#### Minimum inhibitory (MIC) and minimum bactericidal (MBC) concentrations of AgNPs(CHA), viability of microbes upon their incubation with materials and determination of the inhibition zone

The procedure was performed as previously reported [11, 12, 18–22]. Briefly, bacterial strains plated onto trypticase soy agar were incubated at 37 ^ο^C for 18–24 h. Three to five isolated colonies of the same morphological appearance were selected from a fresh agar plate using a sterile loop and transferred into a tube containing 2 mL of sterile saline solution. The optical density at 620 nm was adjusted to 0.1, which corresponded to 10^8^ cfu/mL [11, 12, 18–22]. For the evaluation of MIC, the inoculum size for broth dilution was 5 × 10^5^ cfu/mL. The culture solution was treated with **AgNPs(CHA)** (5–100 μg/mL).

For the evaluation of MBC, the bacteria were initially cultivated in the presence of **AgNPs(CHA)** in broth culture for 20 h. The MBC values were determined in duplicate, by subculturing 4 μL of the broth an agar plate [11, 12, 18–22].

For the evaluation of the viability of microbes on pHEMA, **pHEMA@AgNPs(CHA)_1** and **pHEMA@AgNPs(CHA)_2**, discs of the materials were placed in the test tubes, which contained 5 × 10^5^ cfu/mL of tested bacterial strains [11, 12, 18–22]. The optical densities of the supernatant solutions were then measured to give the % viability of microbes after incubation for 18–24 h.

For the evaluation of IZ, CHA and **AgNPs(CHA)** at 1 and 2 mgr/mL and the disks of pHEMA, **pHEMA@AgNPs(CHA)_1** and **pHEMA@AgNPs(CHA)_2** were placed on the agar surface and the Petri plates were incubated for 20 h.

#### In vitro toxicity against normal human corneal epithelial cells (HCECs)

The procedure was performed as previously reported [11, 12, 18–22]. Briefly, HCECs were seeded in a 24-well plate at a density of 7.5 × 10^4^ cells and after 24 h of cell incubation, **AgNPs(CHA)** and the discs of pHEMA, **pHEMA@AgNPs(CHA)_1** and **pHEMA@AgNPs(CHA)_2** were added into the wells. After 24 h of incubation of the HCECs with the discs, the discs were removed and the culture medium was aspirated and the cells were fixed with 300 μL of 10% cold trichloroacetic acid (TCA). The plate was left for 30 min at 4 °C, washed five times with deionized water, and left to dry at room temperature for at least 24 h. Subsequently, 200 μL of 0.4% (w/v) sulforhodamine B (SRB) (Sigma) in 1% acetic acid solution was added to each well and left at room temperature for 20 min. SRB was removed, and the plate was washed five times with 1% acetic acid before air drying. Bound SRB was solubilized with 1 mL of 10 mM un-buffered Tris-base solution. Absorbance was read at 540 nm.

#### In vivo toxicity of the disks

The brine shrimp assay was performed as previously reported [32]. Briefly, an aliquot (0.1 mL) containing about 6 to 10 nauplii was introduced to each well of a 24-well plate and one disc of pHEMA, **pHEMA@AgNPs(CHA)_1** and **pHEMA@AgNPs(CHA)_2** was added into each well. The final volume of each well is 1 mL with NaCl 0.9%. The brine shrimps were observed at the interval time of 24 h, using a stereoscope. The larvae are considered dead if they do not show any internal or external movement in 10 s of observation. Each experiment was repeated three times.

#### In vivo genotoxicity by *Allium cepa* test

The procedure was performed as previously reported [33]. Briefly, small bulbs (∼1.0–1.5 cm in diameter) of *Allium cepa* were purchased from the local market. Bulbs of *Allium cepa* were placed in test tubes (16 ml) which were filled with water and placed in the incubator at 25 °C, 50–60% humidity and 12 h day lighting/12 h dark for 48 h. The discs of pHEMA, **pHEMA@AgNPs(CHA)_1** and **pHEMA@AgNPs(CHA)_2** were added into the test tubes to incubate the bulbs for 48 h. The roots growing in double distilled water were used as control. In order to evaluate the rate of the cellular division, the microscopic parameter of the mitotic index was determined. All categories were analyzed by counting 1800 cells per concentration (300 cells per slide, a total of six slides).

#### Limitations of the study

While the results of this study are promising, several limitations should be acknowledged. First, the morphological changes in bacteria following treatment with **AgNPs(CHA)** were not assessed using high-resolution imaging techniques such as FESEM due to lack of access to the required instrumentation. Second, although the in vitro cytotoxicity and short-term in vivo toxicity assessments indicate biocompatibility, long-term exposure effects and ocular irritation studies in mammalian models were not conducted. Additionally, the antimicrobial performance was only tested against selected Gram-positive and Gram-negative strains, and further studies are required to evaluate the spectrum of activity, including against resistant clinical isolates. Finally, the release kinetics of silver ions from the hydrogel matrix and their stability under physiological tear film conditions were not explored in depth, which are critical factors for real-world application in contact lens materials.

## Supplementary Information

Below is the link to the electronic supplementary material.

Supplementary file1 (DOCX 2506 KB)

## Data Availability

No datasets were generated or analysed during the current study.

## References

[1] Selvanathan V, Aminuzzaman M, Tan LX, Win YF, Cheah ESG, Heng MH, Tey L-H, Arullappan S, Algethami N, Alharthi SS, Sultana S, Shahiduzzaman, Abdullah H, Aktharuzzaman (2022) Synthesis, characterization, and preliminary in vitro antibacterial evaluation of ZnO nanoparticles derived from soursop (*Annona**muricata* L.) leaf extract as a green reducing agent. J Mater Res Technol 20:2931–2941

[2] Heng MH, Win YF, Cheah ESG, Chan YB, Rahman K, Sultana S, Tey L-H, Wong LS, Djearamane S, Akhtaruzzaman AM (2024) Microwave-assisted green synthesis, characterization, and in vitro antibacterial activity of NiO nanoparticles obtained from lemon peel extract. Green Process Synth 2024:20240071

[3] Chan YB, Aminuzzaman M, Rahman K, Win YF, Sultana S, Cheah SY, Watanabe A, Wong LS, Guha SK, Djearamane S, Rajendran V, Akhtaruzzaman TLH (2024) Green synthesis of ZnO nanoparticles using the mangosteen (*Garcinia**mangostana* L.) leaf extract: comparative preliminary in vitro antibacterial study. Green Process Synth 13:20230251

[4] Rizzello L, Pompa PP (2014) Nanosilver-based antibacterial drugs and devices: mechanisms, methodological drawbacks, and guidelines. Chem Soc Rev 43:1501 24292075 10.1039/c3cs60218d

[5] Eckhardt S, Brunetto PS, Gagnon J, Priebe M, Giese B, Fromm KM (2013) Nanobio silver: its interactions with peptides and bacteria, and its uses in medicine. Chem Rev 113:4708–4754 23488929 10.1021/cr300288v

[6] Pucelik B, Sułek A, Borkowski M, Barzowska A, Kobielusz M, Dąbrowski JM (2022) Synthesis and characterization of size- and charge-tunable silver nanoparticles for selective anticancer and antibacterial treatment. ACS Appl Mater Interfaces 14(13):14981–14996 35344328 10.1021/acsami.2c01100 PMC8990520

[7] Li J, Yuan Z, Liu H, Feng J, Chen Z (2019) Size-dependent tissue-specific biological effects of core–shell structured Fe₃O₄@SiO₂–NH₂ nanoparticles. J Nanobiotechnol 17:124 10.1186/s12951-019-0561-4 PMC6929447 31870377

[8] Shang L, Nienhaus K, Nienhaus GU (2014) Engineered nanoparticles interacting with cells: size matters. J Nanobiotechnol 12:5 10.1186/1477-3155-12-5 PMC3922601 24491160

[9] Meikle TG, Dyett BP, Strachan JB, White J, Drummond CJ, Conn CE (2020) Preparation, characterization, and antimicrobial activity of cubosome-encapsulated metal nanocrystals. ACS Appl Mater Interfaces 12(6):6944–6954 31917545 10.1021/acsami.9b21783

[10] Vidyasagar R, Patel RR, Singh SK, Singh M (2023) Green synthesis of silver nanoparticles: methods, biological applications, delivery and toxicity. Mater Adv 4:1831–1849

[11] Meretoudi A, Banti CN, Raptis PK, Papachristodoulou C, Kourkoumelis N, Ikiades AA, Zoumpoulakis P, Mavromoustakos T, Hadjikakou SK (2021) Silver nanoparticles from oregano leaves’ extracts as antimicrobial components for non-infected hydrogel contact lenses. Int J Mol Sci 22:3539 33805476 10.3390/ijms22073539 PMC8037402

[12] Rossos AK, Banti CN, Raptis PK, Papachristodoulou C, Sainis I, Zoumpoulakis P, Mavromoustakos T, Hadjikakou SK (2021) Silver nanoparticles using eucalyptus or willow extracts (AgNPs) as contact lens hydrogel components to reduce the risk of microbial infection. Molecules 26:5022 34443612 10.3390/molecules26165022 PMC8400931

[13] Duman F, Ocsoy I, Kup FO (2016) Chamomile flower extract-directed CuO nanoparticle formation for its antioxidant and DNA cleavage properties. Mater Sci Eng C 60:333–338 10.1016/j.msec.2015.11.052 26706538

[14] Scalia S, Giuffreda L, Pallado P (1999) Analytical and preparative supercritical fluid extraction of Chamomile flowers and its comparison with conventional methods. J Pharm Biomed Anal 21:549–558 10701421 10.1016/s0731-7085(99)00152-1

[15] Dai YL, Li Y, Wang Q, Niu FJ, Li KW, Wang YY, Wang J, Zhou CZ, Gao LN (2023) Chamomile: a review of its traditional uses, chemical constituents, pharmacological activities and quality control studies. Molecules 28:133 10.3390/molecules28010133 PMC9822300 36615326

[16] Kharaghani D, Dutta D, Gitigard P, Tamada Y, Katagiri A, Phan D-N, Willcox MDP, Kim IS (2019) Development of antibacterial contact lenses containing metallic nanoparticles. Polym Test 79:106034

[17] Khan SA, Lee C-S (2020) Recent progress and strategies to develop antimicrobial contact lenses and lens cases for different types of microbial keratitis. Acta Biomater 113:101–118 32622052 10.1016/j.actbio.2020.06.039

[18] Banti CN, Kapetana M, Papachristodoulou C, Raptopoulou C, Psycharis V, Zoumpoulakis P, Mavromoustakos T, Hadjikakou SK (2021) Hydrogels containing water soluble conjugates of silver(I) ions with amino acids, metabolites or natural products for non-infectious contact lenses. Dalton Trans 50:13712–13727 34636378 10.1039/d1dt02158c

[19] Chrysouli MP, Banti CN, Milionis I, Koumasi D, Raptopoulou CP, Psycharis V, Sainis I, Hadjikakou SK (2018) A water-soluble silver(I) formulation as an effective disinfectant of contact lenses cases. Mater Sci Eng C 93:902–910 10.1016/j.msec.2018.08.061 30274127

[20] Karetsi VA, Banti CN, Kourkoumelis N, Papa-Christodoulou C, Stalikas CD, Raptopoulou CP, Psycharis V, Zoumpoulakis P, Mavromoustakos T, Sainis I, Hadjikakou SK (2019) An efficient disinfectant, composite material SLS@[Zn3(CitH)2] as ingredient for development of sterilized and non-infectious contact lens. Antibiotics 8:213 31703330 10.3390/antibiotics8040213 PMC6963967

[21] Rossos AK, Banti CN, Kalampounias A, Papa-Christodoulou C, Kordatos K, Zoumpoulakis P, Mavromoustakos T, Kourkoumelis N, Hadjikakou SK (2020) pHEMA@AGMNA-1: a novel material for the development of antibacterial contact lens. Mater Sci Eng C 111:110770 10.1016/j.msec.2020.110770 32279741

[22] Milionis I, Banti CN, Sainis I, Raptopoulou CP, Psycharis V, Kourkoumelis N, Hadjikakou SK (2018) Silver ciprofloxacin (CIPAG): a successful combination of antibiotics in inorganic-organic hybrid for the development of novel formulations based on chemically modified commercially available antibiotics. J Biol Inorg Chem 23:705–723 29654371 10.1007/s00775-018-1561-9

[23] Varikooty J, Keir N, Woods CA, Fonn D (2010) Measurement of the refractive index of soft contact lenses during wear. Eye Contact Lens 36:2–5 20009942 10.1097/ICL.0b013e3181c8135f

[24] Caló E, Khutoryanskiy VV (2015) Biomedical applications of hydrogels: a review of patents and commercial products. Eur Polym J 65:252–267

[25] Childs A, Li H, Lewittes D, Dong B, Liu W, Shu X, Sun C, Zhang HF (2016) Fabricating customized hydrogel contact lens. Sci Rep 6:34905 27748361 10.1038/srep34905 PMC5066254

[26] Abdel-Halim ES, Alanazi HH, Al-Deyab SS (2015) Utilization of hydroxypropyl carboxymethyl cellulose in synthesis of silver nanoparticles. Int J Biol Macromol 75:467–473 25697673 10.1016/j.ijbiomac.2015.02.010

[27] Shameli K, Ahmad MB, Zamanian A, Sangpour P, Shabanzadeh P, Abdollahi Y, Zargar M (2012) Green bio-synthesis of silver nanoparticles using *Curcuma**longa* tuber powder. Int J Nanomed 7:5603–5610 10.2147/IJN.S36786 PMC3546626 23341739

[28] Garza-Navarro MA, Aguirre-Rosales JA, Llanas-Vázquez EE, Moreno-Cortez IE, Torres-Castro A, González-González V (2013) Totally ecofriendly synthesis of silver nanoparticles from aqueous dissolutions of polysaccharides. Int J Polym Sci 2013:436021

[29] Rönkkö S, Vellonen K-S, Järvinen K, Toropainen E, Urtti A (2016) Human corneal cell culture models for drug toxicity studies. Drug Deliv Transl Res 9:660–675 10.1007/s13346-016-0330-y PMC5097077 27613190

[30] Liao C, Li Y, Tjong SC (2019) Bactericidal and cytotoxic properties of silver nanoparticles. Int J Mol Sci 20:449 30669621 10.3390/ijms20020449 PMC6359645

[31] Koski C, Sarkar N, Bose S (2020) Cytotoxic and osteogenic effects of crocin and bicarbonate from calcium phosphates for potential chemopreventative and anti-inflammatory applications in vitro and in vivo. J Mater Chem B 8:2048–2062 32064472 10.1039/c9tb01462d

[32] Banti CN, Hadjikakou SK (2021) Evaluation of toxicity with brine shrimp assay. Bio-Protoc 11:e3895 33732784 10.21769/BioProtoc.3895 PMC7952950

[33] Banti CN, Hadjikakou SK (2019) Evaluation of genotoxicity by micronucleus assay in vitro and by Allium cepa test in vivo. Bio-Protoc 9:e3311 33654820 10.21769/BioProtoc.3311 PMC7854110

[34] Ma TH, Cabrera GL, Owens E (2005) Genotoxic agents detected by plant bioassays. Rev Environ Health 20:1–14 15835495 10.1515/reveh.2005.20.1.1

