# Supplementary material for: Biogenic silver nanoparticles from chamomile incorporated in hydrogels for high transparent non-infectiveness contact lenses
Source: J Biol Inorg Chem. 2025 Aug 4;30(6-8):465–83. doi: 10.1007/s00775-025-02121-0 (PMC12675572; doi:10.1007/s00775-025-02121-0)
Supplement: Supplementary file 1 — Supplementary file1 (DOCX 2506 KB) [file 775_2025_2121_MOESM1_ESM.docx]

Supplementary Materials Information

Biogenic Silver Nanoparticles from Chamomile Incorporated in Hydrogels for high transparent non-infectiveness Contact Lenses

Panagiotis K. Raptis^1^, Christina N. Banti^1,^*, Christina Papachristodoulou^2^ and Sotiris.K. Hadjikakou^1,3,^*

^1^ Biological Inorganic Chemistry laboratory, Department of Chemistry, University of Ioannina, 45110 Ioannina, Greece;

^2^ Department of Physics, University of Ioannina, Greece

^3^ University Research Center of Ioannina (URCI), Institute of Materials Science and Computing, Ioannina, Greece

*All correspondence should be addressed to:

Dr. C.N. Banti (Adjunct Lecturer); email: cbanti@uoi.gr

Dr. S.K. Hadjikakou (Professor); e-mail: shadjika@uoi.gr; tel. x30-26510-08374, x30-26510-08362

**Figure S1.** Powder X-Ray Diffraction of **pHEMA@AgNPs(CHA)_1** or **pHEMA@AgNPs(CHA)_2** along with the corresponding ones of **pHEMA** and **AgNPs(CHA)**.

[A] [B]

[C] [D]

**Figure S2.** TGA-DTA Thermogram of [**AgNPs(CHA)** [A] and the corresponding of pHEMA [B], **pHEMA@AgNPs(CHA)_1** [C] and **pHEMA@AgNPs(CHA)_2** [D].

**Figure S3.** ATR-FTIR spectra of pHEMA, **pHEMA@AgNPs(CHA)_1** and **pHEMA@AgNPs(CHA)_2** .


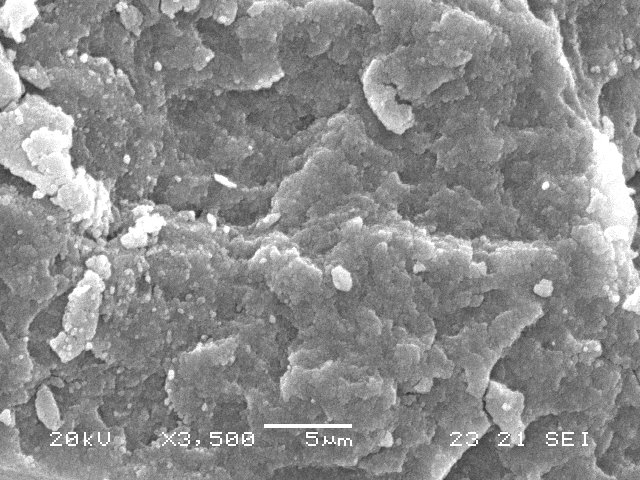


**Figure S4**. SEM image of **AgNPs(CHA).**


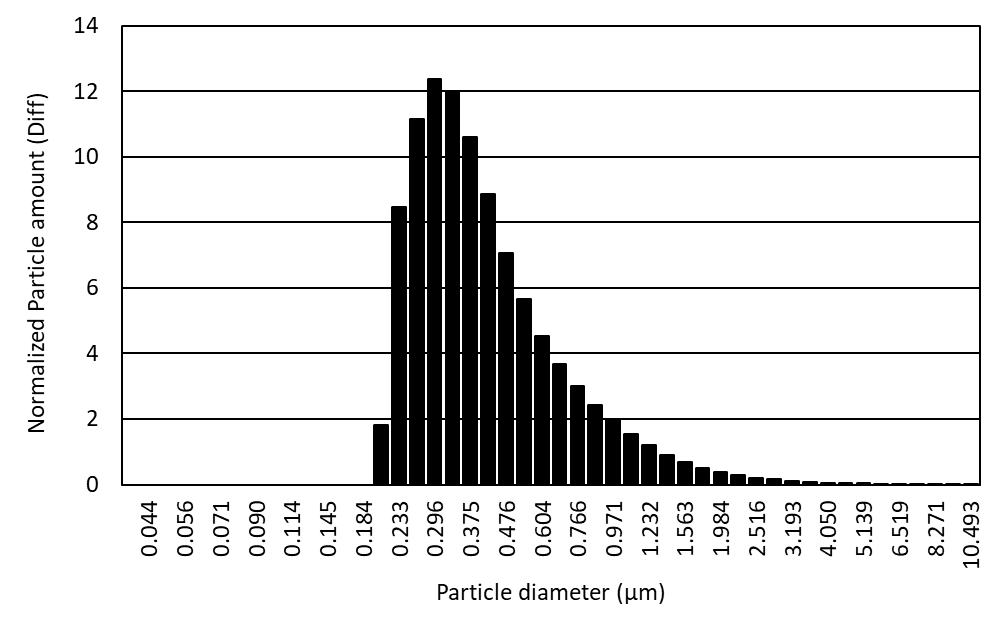


**Figure S5**. DLS analysis for **AgNPs(CHA).**
